# Blood-based biomarkers for immune-based therapy in advanced HCC: Promising but a long way to go

**DOI:** 10.3389/fonc.2022.1028728

**Published:** 2022-10-31

**Authors:** Pil Soo Sung, Isaac Kise Lee, Pu Reun Roh, Min Woo Kang, Jaegyoon Ahn, Seung Kew Yoon

**Affiliations:** ^1^ Department of Biomedicine and Health Sciences, The Catholic University Liver Research Center, College of Medicine, POSTECH-Catholic Biomedical Engineering Institute, The Catholic University of Korea, Seoul, South Korea; ^2^ Division of Gastroenterology and Hepatology, Department of Internal Medicine, College of Medicine, Seoul St. Mary’s Hospital, The Catholic University of Korea, Seoul, South Korea; ^3^ Department of Computer Science and Engineering, Incheon National University, Incheon, South Korea

**Keywords:** hepatocellular carcinoma, immune checkpoint inhibitors, biomarker, circulating tumor DNA, patient outcomes

## Abstract

The introduction of immune checkpoint inhibitors (ICIs) represents a key shift in the management strategy for patients with hepatocellular carcinoma (HCC). However, there is a paucity of predictive biomarkers that facilitate the identification of patients that would respond to ICI therapy. Although several researchers have attempted to resolve the issue, the data is insufficient to alter daily clinical practice. The use of minimally invasive procedures to obtain patient-derived specimen, such as using blood-based samples, is increasingly preferred. Circulating tumor DNA (ctDNA) can be isolated from the blood of cancer patients, and liquid biopsies can provide sufficient material to enable ongoing monitoring of HCC. This is particularly significant for patients for whom surgery is not indicated, including those with advanced HCC. In this review, we summarize the current state of understanding of blood-based biomarkers for ICI-based therapy in advanced HCC, which is promising despite there is still a long way to go.

## Introduction

Hepatocellular carcinoma (HCC) is the most common primary liver cancer and is the sixth most common cancer and the third leading cause of malignancy-associated deaths worldwide (1, 2). HCC primarily occurs after liver cirrhosis, and the most common associated risk factor is chronic hepatitis B virus (HBV) infection; other causes include chronic hepatitis C virus (HCV) infection, alcoholic liver disease, and non-alcoholic fatty liver disease (NAFLD) (3). Liver cancer staging is usually determined by using the 20-year-old Barcelona Clinic Liver Cancer (BCLC) method or its updated versions, and the analysis outcome is used to stratify and allocate HCC patients into appropriate treatment streams (4). According to the BCLC staging system, a considerable number of HCC patients have BCLC stage C HCC, indicating an advanced stage cancer for which curative or locoregional therapies are unsuitable (5, 6). As more than 50% of patients with HCC have a tumor that is too-advanced for curative therapy, HCC represents the second leading cause of death from cancer; with a 3% survival rate at 5 years globally (7). Sorafenib has been used for over ten years as the primary treatment and was the first tyrosine kinase inhibitor (TKI) for patients with advanced HCC. Recently, treatment options for patients with advanced HCC have increased as several novel therapies have gained approval. These include the use of TKIs such as lenvatinib, regorafenib, and cabozantinib and immune-based therapies such as the immune checkpoint inhibitors (ICIs) atezolizumab, nivolumab, and pembrolizumab (8, 9). According to the recent European Association for the Study of the Liver (EASL) guideline, atezolizumab plus bevacizumab or durvalumab plus tremelimumab administration are the preferred first-line treatment in patients with advanced HCC (4, 10, 11).

The liver is characterized by immune tolerance controlled by antigen-presenting cells, which are responsible for the active control of immunogenicity (8). Moreover, during hepatocarcinogenesis, the gradual dysfunction of innate and adaptive immune cells and an increase in the number of immune-regulatory cells contribute to the generation of immunosuppressive tumor microenvironment (TME) (8, 12). M2-polarized tumor-associated macrophages (TAMs), which act as immune suppressors, critically contribute to the immunosuppressive character of the HCC TME (13, 14). Exhausted T cells show upregulated expression of several inhibitory receptors, including programmed cell death protein-1 (PD-1), T cell immunoreceptor with Ig and ITIM domains (TIGIT), T cell immunoglobulin, mucin-domain containing-3 (TIM-3), and lymphocyte activation gene 3 (LAG3), and the effector function of the cells is impaired by transcriptional changes mediated by thymocyte selection-associated HMG BOX (TOX) (15). Blocking such inhibitory receptors with ICIs reinvigorates exhausted T cells and enhances effector function. Over the past ten years, several clinical trials have demonstrated the contribution of ICIs to the improvement of overall survival (OS) of patients with various tumors. Notably, immunotherapies are generally safe and well-tolerated by patients (16); however, under some circumstances, ICI therapy can cause serious adverse events (AEs), resulting in discontinuation and disease hyper-progression in some cases (17). It is critical to identify reliable biomarkers that would facilitate the selection of patients who will be responsive to ICI treatment as well as those likely to suffer serious AEs.

Molecular biomarkers are prevalent in the blood or tissues (18). Several putative biomarkers are being evaluated in clinical trials for ICI therapy against HCC, including programmed death ligand-1 (PD-L1) expression, microsatellite instability (MSI), tumor mutational burden (TMB), DNA damage repair gene alterations, gut microbiome, and various blood biomarkers (16, 19). Among these, markers in blood samples are easily measured and can serve as tools for clinical management, diagnosis, staging, and evaluation of therapeutic response (18). Here, we comprehensively review blood-based biomarkers for the prediction of ICI responses in patients with HCC.

## Currently used immune-based treatments for HCC

### ICI treatment (nivolumab, pembrolizumab, ipilimumab, tremelimumab, and durvalumab)

Nivolumab, the initial humanized IgG4 monoclonal antibody directed against PD-1, reinvigorates the immune response of the host towards cancer cells through the competitive inhibition of PD-1-dependent signaling (20). In patients with advanced HCC recruited to the phase I/II CheckMate-040 study, nivolumab showed an objective response rate (ORR) and disease control rate (DCR) of 15%–20% and 58%–64%, respectively, with an acceptable safety profile (20). Subsequently, the international phase III randomized controlled trial, CheckMate 495, was conducted to determine the efficacy of nivolumab versus sorafenib as first-line monotherapy in patients with advanced HCC (21). The mean OS for patients treated with nivolumab was similar to that for a cohort administered with sorafenib, i.e., 16.4 vs. 14.7 months (HR, 0.85; p = 0.0752), although the predetermined criterion for significance was not met (HR, 0.84; p = 0.0419). When compared with the sorafenib group, the cohort that received nivolumab had a superior ORR, i.e., 7% vs. 15%. A more favorable toxicity profile was obtained using nivolumab, and grade 3–4 AEs associated with therapy were also fewer, i.e., 22% vs. 49% (21).

After the successful demonstration of the safety and anti-tumor activity of pembrolizumab in patients with advanced HCC in the KEYNOTE-224 study (22), a randomized placebo-controlled phase III trial (KEYNOTE-240) assessed the efficacy and safety of pembrolizumab versus best supportive care compared to the placebo in patients with advanced HCC. However, the enhanced OS and progression-free survival (PFS) did not reach the required significance, i.e., 13.9 vs. 10.6 months (p = 0.0238) and 3.0 vs. 2.0 months (p = 0.0022), respectively, when compared with patients administered placebo in sorafenib progressors (23). Another anti-PD-1 antibody, camrelizumab, was also shown to be effective in patients with unresectable HCC. In a phase 2 study with advanced HCC with prior treatment, camrelizumab administration resulted in a 6-month survival rate and response rate of 74.4% and 14.7%, respectively (24).

The checkpoint molecule CTLA-4 also constrains the immune response by restricting the excessive stimulation of effector T cells (25). The human IgG2 monoclonal antibody tremelimumab binds to CTLA-4, which overrides the B7-CTLA-4-mediated downregulation of the T-cell response in the tumor microenvironment. A phase III trial for tremelimumab in patients with advanced HCC reported an ORR of 17.6%, time to progression (TTP) of 6.48 months, and acceptable AEs (26). Ipilimumab is another anti-CTLA-4 antibody that shows superior anti-cancer activity in combination with other drugs (27). In March 2020, a combination of nivolumab and ipilimumab was approved by the FDA for the treatment of patients with advanced HCC who had earlier received sorafenib, which was based on the results of phase I/II CheckMate-040 cohort 4 data (27).

A phase I/II open-label randomized trial investigating combinatorial treatment using durvalumab (anti-PD-L1 monoclonal antibody) plus single-dose tremelimumab in patients with advanced HCC reported positive results (28). In the study, combined therapy with tremelimumab (high priming, 300 mg) plus durvalumab had the longest median OS of 18.7 months, tremlimumab administration alone had a median OS of 15.1 months, durvalumab alone had a median OS of 13.57 months, and the combination of tremelimumab (75 mg) plus durvalumab had the lowest median OS of 11.30 months (28). In a recent open-label phase 3 trial of tremelimumab plus durvalumab, a regimen termed STRIDE (Single 300mg Tremelimumab Regular Interval Durvalumab) was compared with durvalumab alone or sorafenib, for first-line treatment for unresectable HCC patients, STRIDE resulted in the significant improvement of OS when compared with sorafenib (11). Moreover, in the study, durvalumab monotherapy was not inferior to sorafenib as a first-line treatment for unresectable HCC (11). Overall, a very recently published BCLC treatment guideline for unresectable HCC recommends STRIDE regimen as a first line treatment option (4).

### ICI + targeted therapy

Atezolizumab is a humanized IgG1 mAb targeting PD-L1. Bevacizumab, commercially known as Avastin, was one of the first approved angiogenesis inhibitors that were effective in treating breast cancer, non-small-cell lung cancer, cervical cancer, and glioblastoma (29). The pivotal open-label phase III IMbrave150 trial assessed the efficacy of the combined treatment with atezolizumab plus bevacizumab compared to sorafenib therapy as the first-line of treatment in unresectable HCC patients (10). The combination therapy resulted in a significant improvement in the twelve-month OS compared to sorafenib monotherapy (67.2% vs. 54.6%, respectively). Additionally, the median PFS was greater (6.8 months) for the combination therapy relative to sorafenib (4.3 months). The hazard ratio (HR) for disease progression or death was 0.59 (95% CI 0.47–0.76; *p* < 0.001) (10).

Lenvatinib, a multikinase inhibitor used in the first-line treatment for advanced HCC, depletes immunosuppressive TAMs and reverses T cell exhaustion within the tumor microenvironment (30, 31). These actions may maximize the clinical efficacy of PD-1 antibodies in reinstating antitumor responses. Based on this hypothesis, a multicenter, open-label study including 104 patients with advanced HCC treated with a combination of lenvatinib and pembrolizumab was conducted. The study reported a median OS and PFS of 22 and 9.3 months, respectively, and grade 3 or higher AEs were reported in 67% of study participants (32). However, phase 3 LEAP-002 trial investigating pembrolizumab plus lenvatinib versus lenvatinib monotherapy did not meet its dual primary endpoints of OS and PFS as a first-line treatment for patients with unresectable HCC (33).

The phase III COSMIC-312 study reached the primary endpoint, demonstrating a significant improvement in PFS with cabozantinib plus atezolizumab compared to sorafenib in unresectable treatment-naive HCC patients, although a statistically significant benefit was not proved for OS (34). Median PFS was 6·8 months (99% CI 5.6–8.3) in the combination treatment group versus 4·2 months (2.8–7.0) in the sorafenib group (99% CI 0.44–0.91, *p* = 0.0012) (34). The improvement in PFS with cabozantinib plus atezolizumab in this study demonstrates that the combination may benefit patients with unresectable HCC (34). Recently, a trial was designed to assess the efficacy of nivolumab in combination with cabozantinib (group A), or nivolumab plus cabozantinib plus ipilimumab (group B), in 71 patients with advanced HCC randomized into two groups for patients that were treated or untreated with sorafenib (35). The disease control rate for groups A and B were 81% and 83%, respectively, and the median PFS was 5.5 and 6.8 months, respectively. Neither arm achieved the median OS. Grade 3/4 AEs were 42% and 71% in groups A and B, respectively (35).

## Blood-based biomarkers for immune-based treatment in advanced HCC

In individuals with HCC, no biomarker has shown reliable accuracy in the prediction of response to ICIs. The extensive variability of HCC lesions in terms of genomic profile and TME raises concerns over the utility of analyses performed on lone tissue specimens. A key merit of circulating biomarkers is that they can be collected easily and measured following immune-based treatment. Recently, the improved survival and response to therapy with nivolumab has been attributed to a small number of biomarkers due to the facilitation of the evaluation of the expression of PD-L1, favorable alpha-fetoprotein (AFP) responses, inflammatory cytokines, and phenotypes of the peripheral serum mononuclear cells. For ICI + targeted therapy, a recent study also showed that pre-existing immunity (high expression of CD274, T-effector signature, and intratumoral CD8+ T cell density) was associated with better clinical outcomes with atezolizumab + bevacizumab therapy (36). In contrast, reduced responses to the therapy were associated with high regulatory T cell to effector T cell ratio and high GPC3 and AFP expression (36). Currently, efforts to identify blood-based biomarkers are on-going for immune-based combination therapies against advanced HCC. **Table 1** shows the currently available blood-based biomarkers in immune-based therapies in HCC.

**Table 1:** Blood-based biomarkers for immune-based treatment in advanced HCC.

Marker	Characteristic	Evidence	Ref.
AFP PIVKA-II	Traditional blood tumor markers in HCC	AFP concentrations of < 400 µg/L before ICI therapy is associated with increased frequencies of complete or partial responses	36, 37
		PIVKA-II reductions of > 50% were positively correlated with increased OS	38
sPD-L1	Soluble form of PD-L1 protein	High sPD-L1 levels may serve as a negative independent prognostic factor for DFS and OS	39
		High sPD-L1 levels may serve as a prognostic indicator for poor OS outcomes	40
IL-6	A key player of inflammation and cancer cell survival	Pre-treatment elevation of serum IL-6 is a significant predictor of non-response to atezolizumab plus bevacizumab therapy	41
TGF-β	A key player in TME-related immunosuppression	Pre-treatment serum TGF-β titers of < 200 pg/mL were predictive of prolonged OS and PFS for pembrolizumab	8, 42
CD137 (4-1BB)	Activation-induced costimulatory molecule in CD8+ T cells	Longer PFS was observed in patients with high serum CD137 concentrations when compared with those with low concentrations (median, 14.2 vs 4.1 months, p = 0.001) in anti-PD-1 (sintilimab) + bevacizumab biosimilar therapy	43
ADAM9 mRNA	ADAM9: membrane protein associated with cancer invasion	Responders to nivolumab showed a significant decrease in serum ADAM9 mRNA levels	44
KI-67-positive CD4/8+ T cell	Markers for activated T cells	The percentages of Ki-67-positive CD4+ and CD8+ T cells in peripheral blood were higher in responders than in non-responders after nivolumab treatment	45
CD4+ PD-1+ cell	Markers for exhausted T cells	The frequency of peripheral CD4+PD-1+ cells before therapy with tremelimumab was increased in the responders	46
CXCR3+CD8+ effector memory T cells	Markers for functionally preserved CD8 T cells	Associated with objective responses (p = 0.0004 and 0.0255, respectively), PFS (p = 0.00079 and 0.0015, respectively), and irAEs (p = 0.0034 and 0.0125, respectively) in anti-PD-1-treated patients with HCC	47
NLR/PLR	Negative prognostic factor in individuals with HCC	OS was increased in patients with NLR in the lower tertile compared to patients with medium or high NLR tertiles (p = 0.015) An elevated NLR > 4.125 was associated with hyper-progressive disease after nivolumab therapy and reduced survival rate	17, 48
		PLR > or = 300 had reduced OS (6.4 vs. 16.5 months, p < 0.0001) and PFS (1.8 vs. 3.7 months, p = 0.0006).	49
ctDNA	Provides an effective cancer ‘fingerprint’ by liquid biopsy	Significant correlation between ctDNA levels and tumor burden in anti-PD1-treated patients with various cancers Significant correlation between ctDNA and tumor burden in HCC patients treated with atezolizumab plus bevacizumab	50, 51

ADAM9, A disintegrin and a metalloprotease 9; AFP, alpha-fetoprotein; ctDNA, circulating tumor DNA; DFS, disease-free survival; HCC, hepatocellular carcinoma; HPD, hyperprogressive disease; HR, hazard ratio; ICI, immune checkpoint inhibitors; irAEs, immune-related adverse events; MMP-9, matrix metalloproteinase 9; NLR, neutrophils to lymphocytes ratio; OS, overall survival; PFS, progression-free survival; PIVKA, protein induced by vitamin K absence or antagonist; PLR, platelet to lymphocyte ratio; sPD-L1, soluble programmed cell death - ligand 1; sPD-1, soluble programmed cell death protein-1; TGF-β, transforming growth factor – beta

### AFP and PIVKA-II

In general, elevated AFP and protein induced by vitamin K absence or antagonist II (PIVKA-II) titers are negative prognostic factors in individuals with HCC (37). AFP concentrations of <400 µg/L before ICI therapy have been associated with increased frequencies of complete or partial responses as the optimal outcomes (52). Early decreases in serum AFP concentrations have been associated with superior responses to ICI therapy in patients with advanced HCC (38). Another report demonstrated a positive correlation between an AFP reduction > 50% or a PIVKA-II reduction > 50%, and ORR of ICI (anti-PD-1), in HCC (48). In addition, AFP and PIVKA-II reductions of > 50% were positively correlated with increased OS (*p* = 0.003 and 0.006) (48). Nevertheless, data from the CheckMate040 study indicated that even though baseline AFP titers of <400 µg/L reflect prolonged OS compared when compared patients with AFP levels ≥ 400 µg/L, the ORR and DCR for nivolumab therapy remained comparable, irrespective of the APF results (53).

A recent study demonstrated that AFP response at 6 weeks after start of atezolizumab + bevacizumab treatment for advanced HCC is a potential blood biomarker for responses to the therapy (45). The authors derived AFP cutoffs of 75% decrease from baseline at 6 weeks to identify responders (45).

### Soluble PD-L1/PD-1

In the CheckMate 040 trial, investigators assessed multiple biomarkers present in the TME to identify putative associations with the higher response rate for nivolumab in patients with advanced HCC (53). Multiple gene expression signatures for inflammation were assessed in fresh and stored tumor samples from both the dose-escalation and -expansion arms of the trial. The results showed that patients who expressed PD-L1 in their tumor samples had survival benefits, and the median OS for patients with detectable (> 1%) expression of PD-L1 was 28.1 months (95% CI 18.2–N/A), whereas that for patients who did not express PD-L1 (< 1% expression) was 16.6 months (95% CI 14.2–20.2, *p* = 0.032) (53). Notably, although PD-L1 appears to be a reliable indicator of tumor response, the study was limited by several factors. Specifically, the use of an unstandardized cut-off of 1% for PD-L1 expression as a marker of positivity, the temporal and spatial heterogeneity of PD-L1 expression, and the highly complex analytic methods required for analysis may prevent the wide application of the results (14, 39). Therefore, as a single biomarker in HCC, the use of PD-L1 expression as a prognostic marker for response to ICI therapy appears restricted and remains unvalidated. Given the absence of a notable heterogeneity of HCC between tumor samples and between individuals, the appraisal of immunogenicity within a tumor may require a range of biomarkers instead of using PD-L1 expression as a single marker.

Recently, Chinese researchers have evaluated the prognostic impact of sPD-L1, which was a negative independent prognostic factor (disease-free survival (DFS), HR = 2.58, 95% CI 1.14–5.84, *p* = 0.023; OS, HR 1.77, 95% CI 1.01–3.12, *p* = 0.048), whereas sPD-1 was a favorable independent prognostic factor (DFS, HR 0.32, 95% CI 0.14–0.74, *p* = 0.007; OS, HR 0.54, 95% CI 0.30–0.98, *p* = 0.044) in patients with HCC (40). Another report showed that high sPD-L1 levels may serve as a potential prognostic indicator for poor OS outcomes in patients with HCC (54). In a recent Korean study with an analysis of 72 patient samples, the median sPD-L1 and sPD-1 levels were 25.72 and 341.44 pg/mL, respectively. Further, the sPD-1 levels in patients treated with nivolumab as a second-line therapy changed serially, and a reduction of >50% in sPD-1 levels was observed immediately after nivolumab administration. However, in that study, sPD-1 levels were not associated directly with prognosis in patients with advanced HCC (55).

### Cytokines and other serum markers

Interleukin (IL)-6 is produced by various cell types such as tumor cells, stromal cells, and various immune cells in TME. It usually contributes to tumor progression by causing intra- and peritumoral inflammation and promoting angiogenesis (41). A recent study measured 34 plasma proteins in sera from HCC patients treated with atezolizumab plus bevacizumab and identified that elevated plasma IL-6 was a significant predictor of non-response to atezolizumab plus bevacizumab therapy (42). IL8 is a proinflammatory CXC chemokine for neutrophil chemotaxis (41). IL-8 is elevated in various types of malignancies and contributes to tumor angiogenesis and metastasis. A recent study demonstrated that HBV-induced IL-8 promotes HCC metastasis and intrahepatic regulatory T cell accumulation, suggesting the potential negative role of IL-8 in immune-based therapy in HCC (56).

Transforming growth factor-β (TGF-β) is a key player in TME-related immunosuppression and cancer cell circumvention of immune responses (8). The combined delivery of antibodies inhibiting TGF-β and PD-L1 enabled the permeation of T cells into the tumor center and induced potent anti-cancer immune activity (43). In advanced HCC, a robust link exists between TGF-β and exhausted immune signatures (57). Several pertinent serum indicators were assessed in a phase II study which included 29 individuals with unresectable HCC who had undergone treatment with pembrolizumab. Among the serum biomarkers, pre-treatment serum TGF-β titers of <200 pg/mL were predictive of greater OS and PFS (58). Galunisertib, a TGF-β receptor 1 inhibitor, has been evaluated in clinical studies and the results showed an OS of 16.8 months in individuals with advanced HCC in whom pre-therapy AFP titers were < 1.5 × upper normal limit (44).

A recent study further identified soluble levels of CD137 (4-1BB) as one of the blood-based biomarkers for anti-PD-1 (sintilimab) + bevacizumab biosimilar (46). CD137 is an activation-induced costimulatory molecule and its expression on CD8+ tumor-infiltrating lymphocytes reportedly represented a distinct activation state among highly exhausted CD8+ T cells in HCC (59). In addition, a markedly longer PFS was observed in patients with high CD137 concentrations when compared with those with low concentrations (median, 14.2 vs 4.1 months, P = 0.001) (46).

Matrix metalloproteinase 9 (MMP-9) secreted by TAMs has been recently reported to be a potential predictor of immune characteristics and immunotherapeutic responses in HCC (60). ADAMs (a disintegrin and metalloprotease) are membrane proteins containing both protease and adhesion domains and thus may be potentially important in cancer invasion and metastasis (61). The ADAM9 mRNA levels in blood samples derived from patients with advanced HCC revealed that among four patients treated with nivolumab therapy, two exhibited a clinical response and showed a significant decrease in serum ADAM9 mRNA levels, whereas the other two patients showed no response to nivolumab and no change in ADAM9 mRNA levels. Although the sample size was small, the results of the present study suggested that ADAM9 mRNA might serve as a predictive biomarker for clinical responses to immunotherapy (61).

### Circulating immune cells

Recently, our group reported that the percentages of Ki-67-positive CD4+ and CD8+ T cells in peripheral blood were higher in responders than in non-responders after nivolumab treatment (39). The frequency of CD4+PD-1+ cells within serum mononuclear cells before therapy with tremelimumab was increased in the responders (62). Decreased serum B cell PD-1 expression at baseline and PD-L1 expression in monocytes following therapy was linked with the disease control in 16 individuals with HCC, who received nivolumab (63).

A recently identified single-cell peripheral immune signature provides promising non-invasive biomarkers for the early detection of HCC and early assessment of anti-PD-1 immunotherapy efficacy in patients with advanced HCC (64). Single-cell analyses using cytometry by time of flight (CyTOF) identified CXCR3+CD8+ effector memory T cells and CD11c+ antigen-presenting cells as being associated with objective responses (p = 0.0004 and 0.0255, respectively), PFS (p = 0.00079 and 0.0015, respectively), and immune-related adverse events (irAEs) (p = 0.0034 and 0.0125, respectively) in anti-PD-1-treated patients with HCC. Type-1 conventional dendritic cells were also identified as the specific antigen-presenting cells associated with the immunotherapy response, whereas two immunosuppressive CD14+ myeloid clusters were linked to reduced irAEs. Another recent report analyzed CXCR3+CD8+ effector memory T cells and showed cell–cell interactions specific to the response to immunotherapy vs. irAEs (65). The anti-PD-1 and anti-TNFR2 combination led to uncouple the efficacy of ICI and irAEs of it, resulting in enhanced response without increased irAEs, in a murine HCC model (65).

### NLR/PLR

Regardless of the underlying causes associated with HCC, inflammation remains a major factor, especially because the evolution of hepatic fibrosis to neoplasia may rely on several intra-hepatic proinflammatory cascades (14, 66). In general, adverse survival statistics and the cancer progression is frequently associated with systemic inflammation (67). Neutrophils may exhibit phenotypic plasticity and can exist both under tumor-promoting and tumor-suppressing states. Tumor-associated neutrophils (TANs) contributes to tumor progression by mitigating antitumor immunity (49). The proinflammatory cytokine IL-17 recruits TANs to the TME of HCC (49). TANs also have additional immunosuppressive functions by the recruitment of regulatory T cells and TAMs to the HCC TME (68). TANs and peripheral blood neutrophils produce CCL2 and CCL17 chemokines, and these recruit macrophages into the TME of HCC (68). A recent report suggested that neutrophils hamper the efficacy of ICIs, especially in non-alcoholic steatohepatitis (NASH)-induced HCC (69). The neutrophils to lymphocytes ratio (NLR) may serve as indicators of systemic inflammation, and the ratio is increased in individuals with HCC, owing to relative increases and decreases in neutrophils and lymphocytes, respectively (70). The association between an elevated NLR and adverse clinical outcomes in individuals with HCC has been demonstrated consistently for several treatment options for HCC, e.g. resection, transplantation, radiotherapy, and TACE (47, 71–73).

NLR has shown promise as a prognostic factor in individuals with advanced HCC treated with nivolumab. In contrast to patients with an NLR ≥ 5, an NLR < 5 before and after therapy was associated with enhanced OS of 23 vs. 10 months; (*p* = 0.004), and 35 vs. 9 months (*p* < 0.0001), respectively (74). Another study using patient data from the Checkmate 040 trial demonstrated that OS was increased in patients with NLR in the lower tertile compared to patients with medium or high NLR tertiles (*p* = 0.015) (53). Furthermore, a multi-center study conducted in Korea demonstrated that an elevated NLR > 4.125 was associated with HPD after nivolumab therapy and reduced survival rate (17). Additionally, the baseline and treatment kinetics for the NLR are effective prognostic indicators in nivolumab-treated patients with HCC (75). During treatment, the NLR increased rapidly in patients with hyperprogressive disease (HPD) (75). In addition, a recent study showed that patients treated with various ICIs and ICI-based combination therapies with an NLR ≥ 5 had reduced OS (7.7 vs. 17.6 months, *p* < 0.0001), reduced PFS (2.1 vs. 3.8 months, *p* = 0.025), and decreased ORR (12% vs. 22%, *p* = 0.034), suggesting that systemic inflammation indicated by NLR is an independent negative prognostic factor in patients with HCC undergoing ICI therapy (76).

The platelet to lymphocyte ratio (PLR) is an additional potential prognostic ranking method, with an increased value indicating a relative rise in the platelet count and a fall in lymphocyte numbers. The former occurs frequently in individuals with HCC and is indicative of portal hypertension, as observed in a study of individuals with late-stage HCC receiving ICI therapy (74). Another study showed that patients with PLR > or = 300 had reduced OS (6.4 vs. 16.5 months, *p* < 0.0001) and PFS (1.8 vs. 3.7 months, *p* = 0.0006). In that study, NLR emerged as an independent prognostic factor for OS in univariate and multivariate analysis (HR 1.95, *p* < 0.001; HR 1.73, *p* = 0.002, respectively) and the PLR remained an independent prognostic factor for both OS and PFS in multivariate analysis (HR 1.60, *p* = 0.020; HR 1.99, *p* = 0.021) (76).

The prognostic nutritional index (PNI) is an immune-nutritional indicator and is calculated from the serum albumin (ALB) level and the peripheral blood and lymphocyte (LYM) count (77). A recent study defined the inflammation‐immunity‐nutrition score (IINS), which was simply based on highly sensitive C-reactive protein (hsCRP), LYM, and ALB (50). HCC Patients with low IINS had longer OS and PFS in response to anti-PD-1, suggesting that IINS may serve as an independent prognostic factor for HCC patients treated with anti‐PD‐1 therapy (51).

Although these preliminary observations hold promise, the inflammatory condition inexorably oscillates during pathological evolution and advance, and therefore the NLR and PLR will change based on the timing of specimen acquisition (67). Generally, these relationships require verification before their implementation in clinical practice through additional analysis in individuals with HCC.

### Circulating tumor DNAs

Recently, liquid biopsy potentially offers a non-invasive tool for monitoring and diagnosis for cancer patients. The application of the method has been encouraging for early diagnosis, determination of residual disease, and decision-making to facilitate systemic treatment for HCC (78). Regarding the choice of liquid biopsy analytes that may be used in HCC, circulating tumor DNA (ctDNA) has made an invaluable contribution to the prognosis and monitoring of disease (79). Cell-free DNA (cfDNA) contains only a small proportion of ctDNA, which demands the application of highly sensitive and reliable methods of detection. Point mutations are determined by either droplet digital PCR (ddPCR) or by NGS-based sequencing (79). Subsequent to tumor cell apoptosis and necrosis, ctDNA can be found in the blood stream of cancer patients (80) and provides an effective cancer ‘fingerprint’ as it has the tumor’s molecular characteristics. ctDNA can be differentiated from normal circulating DNA using various markers, including mutation signatures (80, 81) and epigenetic alterations (82). The variation in the concentration of ctDNA in the blood provides a quantitative measure, whereas assessment of gene mutations, changes in DNA copy number, and methylation profiles provide a qualitative assessment of the disease condition. In some situations, ctDNA analysis may help identify mutations that were not obtained from a single tumor biopsy (83). Both genomic and epigenetic biomarker modifications in HCC are important factors to consider in recurrence monitoring and precision oncology (84). The results highlight the value of incorporating ctDNA analysis for the diagnosis and prognosis of patients with HCC. Our recent study using targeted NGS technique identified at least one pathogenic variant of two major HCC driver genes (TP53 and CTNNB1), including 16 variants of TP53 and nine variants of CTNNB1 in 65% HCC patients (13/20) (81).

To relate ctDNA to immunotherapy for cancers, a recent study demonstrated that significant correlation between ctDNA levels and tumor burden in anti-PD1-treated patients with various cancers (85). From the patients enrolled in the phase 1b clinical trial of atezolizumab plus bevacizumab, researchers were able to identify a statistically significant correlation between ctDNA and tumor burden (p < 0.03). Of this sub-cohort, patients who achieved a complete response, partial response, stable disease state, and disease progression, ctDNA was undetectable in 70%, 27%, 9% and 0% of these groups, respectively. Furthermore, in cases where ctDNA was not detectable during their treatment period, the patients thereafter experienced a longer PFS (86). Another very recent study using patient cohort treated with camrelizumab plus apatinib demonstrated that patients who were ctDNA positive after adjuvant therapy presented a trend of shorter RFS than those who were ctDNA negative (87). The role of methylated ctDNA in predicting immunotherapy responses is studied in other cancer types such as colorectal cancer (CRC). For example, in four case studies of methylated SEPTIN9 gene (mSEPT9) as a marker of response to immunotherapy in metastatic CRC on four patients, the marker shows that a decrease in ctDNA levels is indicative of a tumor response to immunotherapy while an increase in ctDNA levels corresponds to tumor progression in response to immunotherapy (88, 89). A similar study that focuses on HCC, however, has yet to be found. Another study that looked at predicting immunotherapy responses in HCC but with a methylated RNA molecule (SNRPC) instead, showed a similar pattern in that high-SNRPC groups showed no response to anti-PD1 therapy while low-SNRPC groups showed more patients responding to immune checkpoint inhibitor therapy (90).

TMB, an emergent determinant of immunotherapy responses, is a measure of the total number of somatic non-synonymous mutations per mega-base in the tumor, and its levels between ctDNA and tumor tissues were found to be consistent. Consequently, analysis of blood samples for TMB prior to immunotherapy in advanced primary liver cancer can be beneficial in predicting the responses (91). However, a recent study using 121 HCC blood samples found that mutational analysis ctDNA was associated with a response to systemic TKI treatment, not with ICI treatment (80).

## Limitations and future perspectives

Currently, blood-based biomarkers are newly identified and validated in immune-based treatments for the various cancer types, although the researches have been mostly focusing on immunogenic tumors such as malignant melanoma or lung cancer for which ICIs are being most actively used. Hopefully, soon, blood-based biomarkers including ctDNA may be approved for predicting of responses to ICIs in these types of immunogenic cancers. However, because each type of cancer has its own immune TME and cytokine milieu, a more complex blood-based biomarker discovery may be needed for other types of heterogeneous tumors. Although several studies have been conducted to identify predictive biomarkers to enable the stratification of patients who could benefit from ICI treatment in HCC, few have been prospectively validated and none have resulted in the rewriting of current clinical guidelines or entered clinical practice. Here, we have summarized the progress of immunotherapies for HCC in recent years, with a particular emphasis on predictive biomarkers. However, as immunotherapy development for HCC is still in its infancy, basic research and clinical trials exploring the predictive efficacy of immunotherapeutic biomarkers are still limited, and it is not yet possible to determine which biomarker(s) can effectively predict the efficacy of immunotherapy. Although evaluation of human tissues using various technologies is now routine for the derivation of biomarkers, the utility of straightforward instruments to obtain prognostic data, and which are easily accessible from general blood samples should not be discounted, as they may represent a simpler and more widely available option in daily clinical settings. To address the issue of low concentrations of biomarkers in blood, platforms such as the SomaScan, are able to measure ~7000 proteins in a drop of serum simultaneously, leading the way for novel protein biomarker discoveries (92). One more thing to consider is irAEs. A recent report demonstrated that development of low-grade irAEs was associated with favorable responses for HCC patients treated with ICIs (93). Because there are no reliable biomarkers for irAEs in HCC either, clinicians should be vigilant for detection of irAEs when treating HCC patients with ICIs or combination therapy.

Regardless of the potential value of using ctDNA as a biomarker for diagnosis and treatment responses, there are several limitations associated with its current use. Specifically, in the early stages of tumorigenesis, the levels of ctDNA in the blood are extremely low, which can hinder the early diagnosis of HCC. Further, there is currently no standardized methodology associated with sample collection, preparation, and data analysis. The current method cannot adequately capture spatial tumor heterogeneity, which is indicative of clonal differences within or across tumor metastases (94–96). To resolve these issues, a combinatorial and/or multiparametric process is required to enhance the sensitivity and specificity of using ctDNA as a putative HCC biomarker. The use of blood-based biomarkers including ctDNA to determine the efficacy of immunotherapy against HCC necessitates additional, well-controlled clinical trials, so that the value and clinical relevance of such research endeavors can be realized.

## Conclusion

Recent advances in research techniques, such as NGS, scRNA sequencing, and artificial intelligence, should facilitate a more comprehensive understanding of the various com-ponents of the TME and their interactions in HCC. Moreover, recent unsatisfactory out-comes in immune-based treatment in advanced HCC urges clinicians to identify blood-based biomarkers for favorable responses to such treatments. Future research should focus on the identification of blood-based protein and cell-free nucleic acid biomarkers for immune-based therapy in HCC.

## Author contributions

Conceptualization, PS and JA; methodology, PS; data curation, PS; writing—original draft preparation, IL, PR, and MK; writing—review and editing, PS and JA; supervision, JA and SY; project administration, PS, JA and SY; funding acquisition, PS and JA; All authors have read and agreed to the published version of the manuscript.

## Funding

The Basic Science Research Program is supported by the Basic Science Research Program through an NRF grant funded by the Ministry of Science and ICT (NRF-2019R1A2C3005212 to JA). Research Fund of Seoul St. Mary’s Hospital of The Catholic University of Korea (to PS).

## Conflict of interest

The authors declare that the research was conducted in the absence of any commercial or financial relationships that could be construed as a potential conflict of interest.

## Publisher’s note

All claims expressed in this article are solely those of the authors and do not necessarily represent those of their affiliated organizations, or those of the publisher, the editors and the reviewers. Any product that may be evaluated in this article, or claim that may be made by its manufacturer, is not guaranteed or endorsed by the publisher.
